# A high-density SNP genetic map consisting of a complete set of homologous groups in autohexaploid sweetpotato (*Ipomoea batatas*)

**DOI:** 10.1038/srep44207

**Published:** 2017-03-10

**Authors:** Kenta Shirasawa, Masaru Tanaka, Yasuhiro Takahata, Daifu Ma, Qinghe Cao, Qingchang Liu, Hong Zhai, Sang-Soo Kwak, Jae Cheol Jeong, Ung-Han Yoon, Hyeong-Un Lee, Hideki Hirakawa, Sachiko Isobe

**Affiliations:** 1 Kazusa DNA Research Institute, Japan; 2 Kyushu Okinawa Agricultural Research Center, National Agriculture and Food Research Organization, Japan; 3 Chinese Academy of Agricultural Sciences, China; 4 China Agricultural University, China; 5 Korea Research Institute of Bioscience & Biotechnology, South Korea; 6 Rural Development Administration, South Korea

## Abstract

Sweetpotato (*Ipomoea batatas*) is an autohexaploid species with 90 chromosomes (2n = 6x = 90) and a basic chromosome number of 15, and is therefore regarded as one of the most challenging species for high-density genetic map construction. Here, we used single nucleotide polymorphisms (SNPs) identified by double-digest restriction site-associated DNA sequencing based on next-generation sequencing technology to construct a map for sweetpotato. We then aligned the sequence reads onto the reference genome sequence of *I. trifida*, a likely diploid ancestor of sweetpotato, to detect SNPs. In addition, to simplify analysis of the complex genetic mode of autohexaploidy, we used an S1 mapping population derived from self-pollination of a single parent. As a result, 28,087 double-simplex SNPs showing a Mendelian segregation ratio in the S1 progeny could be mapped onto 96 linkage groups (LGs), covering a total distance of 33,020.4 cM. Based on the positions of the SNPs on the *I. trifida* genome, the LGs were classified into 15 groups, each with roughly six LGs and six small extra groups. The molecular genetic techniques used in this study are applicable to high-density mapping of other polyploid plant species, including important crops.

Genetic maps, or DNA marker linkage maps, are essential tools for genomics and genetics research, e.g., map-based cloning, comparative map analysis, and anchoring genome sequences to chromosomes^1^,^2^. High-density genetic maps saturated with DNA marker loci are especially helpful for these studies. The types of DNA markers used for map construction affect the density and accuracy of the resulting maps. Simple sequence repeat (SSR) markers, or so-called microsatellite markers, were previously the most popular DNA markers due to their usefulness for multi-allelic detection, high-transferability across species, and flexibility, as they can be used with various detection systems^3^. Recent advances in DNA analysis methods and sequencing throughput via next-generation sequencing (NGS) technology have enabled large numbers of single nucleotide polymorphism (SNP) markers to be developed in a single experiment^4^. These SNP markers, as well as SSR markers, have facilitated the establishment of ultra high-density genetic maps in many plant species^1^,^2^. SNPs have several advantages over SSRs: they are the most abundant DNA polymorphisms in the genome and can therefore be utilized in readily available, cost-effective genotyping methods, e.g., genotyping by sequencing (GBS)^5^ and restriction site-associated DNA sequencing (RAD-Seq)^6^ based on NGS technology^7^.

The genome structure of the target species is another important factor for choosing a map construction strategy. Polyploidy, i.e., the presence of multiple sets of chromosomes in a single plant, is commonly observed in the plant kingdom. Polyploid plant species are often used as crops because of their larger plant sizes and yields due to genome multiplication, which can lead to heterosis, gene redundancy, loss of self-incompatibility, and gains in asexual reproduction^8^. Therefore, constructing genetic maps for polyploid species is important for identifying beneficial trait loci and performing genome-based breeding. Polyploid plants can be allopolyploids or autopolyploids. In allopolyploids, chromosome pairings generally occur between homologous chromosomes, but not between homeologs, with a few exceptions^9^. Therefore, the manner of inheritance is expected to be similar to that in diploids, i.e., Mendelian inheritance. By contrast, in autopolyploids, one chromosome pairs with either homologous chromosome counterpart, resulting in a complex inheritance pattern. In the progeny of autotetraploid crops including potato (*Solanum tuberosum*), rose (*Rosa hybrida*), alfalfa (*Medicago sativa*), and blueberry (*Vaccinium corymbosum*), theoretical models of linkage analysis have been thoroughly investigated^10^,^11^,^12^,^13^, and TetraploidMap software is available to construct linkage maps for autotetraploid species^14^. On the other hand, in autohexaploids, e.g., sweetpotato (*Ipomoea batatas*), the genetic model is complex due to the 13 possible allele combinations (Supplementary Table S1). Among these, simplex (e.g., AAAAAA × AAAAAa) and double-simplex markers (AAAAAa × AAAAAa) show simple disomic-like segregation patterns of 1:1 and 1:2:1, respectively, in the progeny (Supplementary Table S1). For linkage analysis, simplex markers are used to construct framework linkage maps, but double-simplex markers, together with duplex (AAAAAA × AAAAaa) and triplex markers (AAAAAA × AAAaaa), are employed to identify homologous groups (HGs)^15^,^16^.

Sweetpotato is an autohexaploid crop with 90 chromosomes (2n = 6x = 90), as mentioned above. Sweetpotato is used worldwide for vegetable and staple food production, as well as food processing starch production. However, the inheritance patterns of agronomically important traits in this crop are extraordinary complex due to its autohexaploidy and large chromosome number. Therefore, genetic maps are needed for efficient breeding. Several genetic maps for sweetpotato based on random amplified polymorphic DNA (RAPD), SSR, amplified fragment length polymorphism (AFLP), and transposon-insertion polymorphism have been reported to date^15^,^16^,^17^,^18^,^19^,^20^. In addition, homologous groups (HGs) have been identified via anchoring with double-simplex, duplex, and triplex markers^15^,^16^. However, these maps are considered to be incomplete, as they contain many short linkage groups (LGs) consisting of a few DNA markers, and small numbers of anchor markers were used to identify HGs.

Three strategies can be utilized to overcome this situation, which have several advantages over previous methods: (1) SNP markers can be used to increase the number of segregated genetic loci; (2) the genome sequence of *I. trifida*, a likely diploid ancestor of sweetpotato^21^, can be used as a reference to detect SNPs and to identify HGs; and (3) an S1 mapping population produced from self-pollination of a single parent can be used to simplify the complex genetic mode of autohexaploidy (Supplementary Table S1). In the current study, to explore these possibilities and establish a high-density genetic map for sweetpotato, we employed double-digest RAD-Seq (ddRAD-Seq) technology^22^, in which genomic DNA is digested with two different restriction enzymes instead of a single nuclease, as used in the original RAD-Seq protocol^6^. Using our recently established analytic pipeline for ddRAD-Seq^23^, we successfully produced the first high-density genetic map for sweetpotato.

## Results

### ddRAD-Seq of an S1 mapping population of cv. Xushu 18

We generated an S1 mapping population (n = 142) via self-pollination of the sweetpotato cultivar, Xushu 18. We used genomic DNA extracted from individuals of this population to construct ddRAD-Seq libraries using two combinations of restriction enzymes, *Pst*I-*Msp*I (PM) and *Pst*I-*Eco*RI (PE). Paired-end sequencing data, as well as single-read sequences, were obtained from the libraries. Approximately 1.8 million (M), 1.2 M, and 1.9 M high-quality reads per line were obtained after trimming adapters and low-quality sequences from paired-end data from the *Pst*I-*Msp*I library (PM-PE) and the *Pst*I-*Eco*RI library (PE-PE) and from single-read data obtained from the *Pst*I-*Msp*I library (PM-SR), respectively (Supplementary Table S2). The reads were mapped onto the reference genome sequence of *I. trifida*, ITR_r1.0, with mapping alignment rates of 73.4% (PM-PE), 78.6% (PE-PE), and 75.5% (PM-SR) (Supplementary Table S2). Of the entire reference sequence (512,990,885 bases), 2,675,332 (0.5%), 1,901,821 (0.4%), and 1,897,014 bases (0.4%) were covered at a depth of ≥10 reads in the PM-PE, PE-PE, and PM-SR libraries, respectively (Supplementary Table S2). As a result, 5,015,871 bases (1.0%) from the reference genome were covered by ≥10 reads from the three libraries. We used the combined data from the three libraries for subsequent analysis.

### SNP calling and selection of double-simplex SNP loci

When sequence reads from the six genomes of hexaploid sweetpotato were mapped onto the haploid genome sequence of *I. trifida*, we assumed that the SNP loci would show six different genotypes: AAAAAa, AAAAaa, AAAaaa, AAaaaa, Aaaaaa, and aaaaaa, where “A” and “a” represent alleles identical to and different from the *I. trifida* alleles, respectively. The AAAAAA genotype would not be identified among SNP loci due to the lack of sequence differences between the two species. Hereafter, “A” and “a” are referred to as REF (reference) and ALT (alternative) alleles, respectively. In addition, the frequency of ALT alleles for each SNP locus is referred to as the ALT allele frequency (AAF), which was calculated with the following formula:

(Number of reads of ALT alleles)/(Number of reads of REF and ALT alleles).

Therefore, theoretical AAFs of the six types should be present in the following ratios: 0.167 (=1/6: AAAAAa), 0.333 (=2/6: AAAAaa), 0.500 (=3/6: AAAaaa), 0.667 (=4/6: AAaaaa), 0.833 (=5/6: Aaaaaa), and 1.000 (=6/6: aaaaaa), together with 0.000 (=0/6: AAAAAA). Indeed, for example, AAF for the 237,861^st^ position in Itr_sc000310.1, at which numbers of reads of REF and ALT alleles across the 142 S1 lines were 17,391 and 5,236, respectively, was calculated to be 0.231 (=5,236/[17,391 + 5,236]).

**Figure 1:**
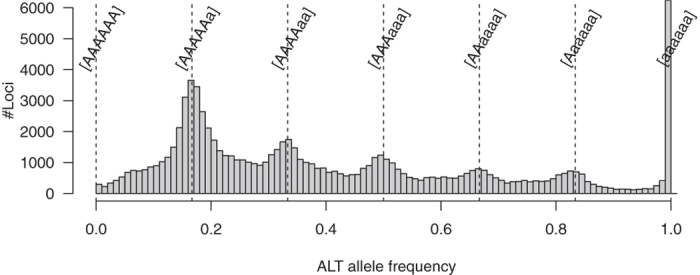
Distribution of ALT allele frequency in the S1 mapping population representing the parental line, Xushu 18. Vertical dashed lines indicate frequencies of 0.000 (0/0), 0.167 (=1/6), 0.333 (=2/6), 0.500 (=3/6), 0.667 (=4/6), 0.833 (=5/6), and 1.000 (=6/6).

Based on the sequence alignment data, 94,361 SNP candidate loci were identified after filtering using two criteria: (i) depth of coverage ≥10 for each S1 line and (ii) proportion of missing data <0.25 for each locus. Since we used only double-simplex markers (AAAAAa × AAAAAa or Aaaaaa × Aaaaaa) for subsequent linkage analysis, further filtering was required to exclude double-duplex (AAAAaa × AAAAaa and AAaaaa × AAaaaa) and double-triplex loci (AAAaaa × AAAaaa). We then calculated the AAFs for each locus. As expected, the distribution pattern of the AAFs exhibited six peaks, with values of 0.167 (=1/6), 0.333 (=2/6), 0.500 (=3/6), 0.667 (=4/6), 0.833 (=5/6), and 1.000 (=6/6) (Fig. 1). We selected 29,701 (AAAAAa × AAAAAa) and 6,889 (Aaaaaa × Aaaaaa) double-simplex loci for further analysis.

Subsequently, we determined the genotypes for each individual for the 36,590 (29,701 + 6,889) SNPs. In the “AAAAAa × AAAAAa” double-simplex SNPs, AAFs of 0.000 (AAAAAA), 0.167 (AAAAAa), and 0.333 (AAAAaa) were expected to segregate at a ratio of 1:2:1 in the S1 progeny. However, it was difficult to distinguish between the AAAAAa and AAAAaa genotypes because numbers of reads in each individual were insufficient to differentiate AAFs of 0.167 and 0.333 significantly. Therefore, we defined an AAF of 0 as indicating homozygous REF alleles and AAF > 0.000 as indicating “not homozygous” REF alleles, with an expected segregation ratio of 1:3, such as dominant loci. We applied the same strategy to the “Aaaaaa × Aaaaaa” double-simplex candidates and determined that AAF of 1.000 indicates homozygous ALT alleles, whereas AAF <1.000 indicates “not homozygous” ALT alleles, with an expected segregation ratio of 1:3. We selected a subset of segregation data fitting the expected ratio via Chi-square tests (*P* ≥ 0.01).

### Grouping and ordering of double-simplex SNP loci

To construct a genetic map, we grouped 28,516 double-simplex SNPs showing a 1:3 segregation ratio in the S1 progeny using the “group” command in the OneMap program. Using the criterion LOD value = 3–10, we obtained 1–105 groups with at least 20 SNPs (Supplementary Fig. S1). Among these, groups with a LOD value of 7 were chosen for further analysis, because, using this criterion, 28,091 SNPs (98.5%) were divided into 98 groups, a number remarkably close to the expected value of 90, i.e., the number of chromosomes in sweetpotato.

**Figure 2:**
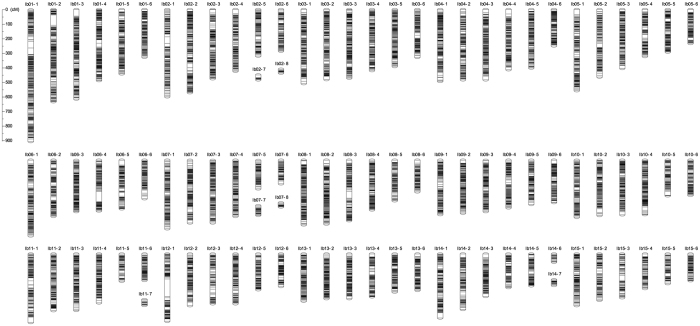
Genetic map of sweetpotato. Detailed information about the map and SNPs is shown in Supplementary Table S3 and is also available at http://sweetpotato-garden.kazusa.or.jp/.

We performed locus ordering within a group with the OneMap ordering command to generate 98 LGs consisting of the 28,087 SNP loci, with a total length of 83,678 cM. The average number of mapped loci on a single LG was 286.6 loci/LG, and the average map length was 853.9 cM/LG. Since genotyping errors and missing data are frequently encountered when genotyping via a sequencing strategy^7^, 1.3% inherent errors and 2.2% missing of the 3.99 M data points (28,087 SNPs across the 142 S1 lines) data were corrected and imputed, respectively, using a map-based imputation algorithm implemented with the Maskov program^24^. The resulting error-reduced imputed data were again subjected to grouping and ordering analysis with OneMap. As a result, we obtained a genetic map consisting of 96 LGs, including 28,087 SNPs in 15,634 co-segregation bins, covering a total distance of 33,020.4 cM (Fig. 2, Table 1, Supplementary Table S3). The mean length and number of mapped SNPs of a single LG were 344.0 cM (ranging from 26.3 to 904.5 cM) and 292.6 SNPs (19–692 SNPs), respectively.

The SNPs on the genetic map were functionally annotated (Supplementary Table S4), because, simultaneously, these SNPs were physically mapped on the reference sequences of the *I. trifida* genome^21^, on which 62,407 genes that occupies 12.5% of the genome were predicted. A total of 24,732 SNPs (88.1%) were in gene regions, while the other 3,236 (11.5%) were in intergenic sequences. The remaining 119 (0.4%) were not assigned. Of the gene SNPs, 146 (0.5%) and 5,847 (20.8%) were putative loss-of-function and non-synonymous SNPs, respectively. The proportions of the SNP effects in the three independent libraries were similar (Supplementary Table S4).

### Identification of HGs from the genetic LGs

**Figure 3:**
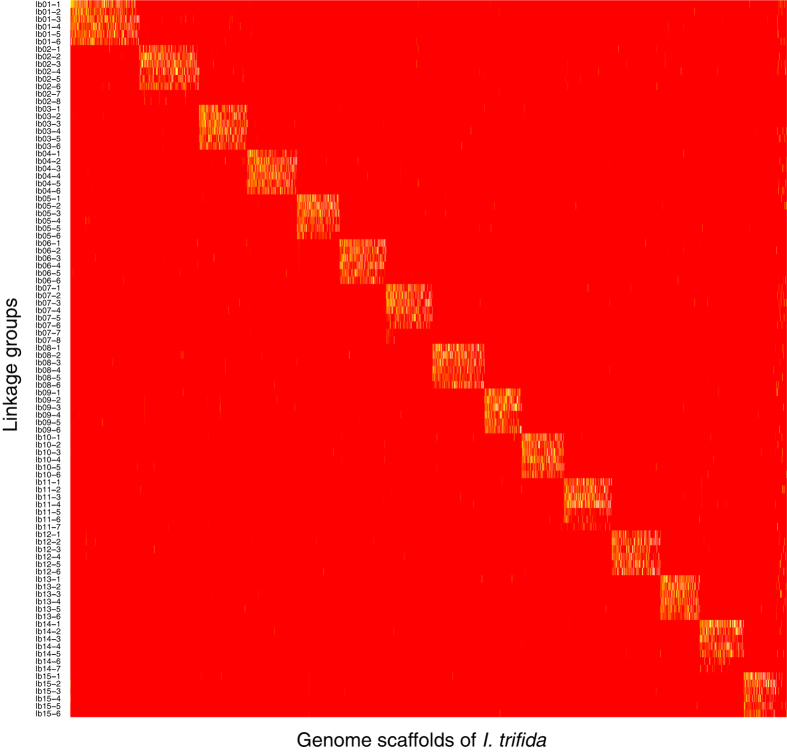
Homologous groups based on physical mapping of the linkage groups onto the *I. trifida* genome scaffolds. Presence and absence of assignments of SNPs on the scaffolds are indicated by yellow and red intersections, respectively, between the linkage groups (vertical axis) and genome scaffolds (horizontal axis).

We reasoned that the 96 LGs belonged to 15 HGs corresponding to the basic chromosome number of sweetpotato. To validate this hypothesis, we assigned the LGs onto the genome sequence of *I. trifida* (ITR_r1.0). The 28,087 SNPs on the 96 LGs were positioned onto 2,889 genome scaffolds covering 236.5 Mb (46.1%) of the total length of ITR_r1.0 (513.0 Mb). Each LG was assigned to an average of 81.6 scaffolds, ranging from 7 to 157. Of the 2,889 *I. trifida* scaffolds, 194 were assigned to six LGs (probably belonging to the same HG), as expected, while 2,657 and 38 scaffolds were assigned to fewer and more than six LGs, respectively. Based on the scaffolds assigned to multiple LGs, the 96 LGs were grouped into HGs (Fig. 3, Supplementary Table S5), and the HGs and LGs were numbered according to map length. Eleven HGs (Ib01, Ib03, Ib04, Ib05, Ib06, Ib08, Ib09, Ib10, Ib12, Ib13, and Ib15) included six LGs, whereas three HGs (Ib02, Ib07, and Ib11) consisted of six main LGs and small extra LGs. For example, Ib14 comprises five main groups and two small groups. Therefore, the 96 LGs were successfully grouped into 15 HGs, as expected.

## Discussion

To the best of our knowledge, this is the first report describing a high-density SNP linkage map for the autohexaploid sweetpotato, *I. batatas*, which was produced using ddRAD-Seq analysis, a high-throughput genotyping strategy. In the past decade, several genetic maps for sweetpotato have been constructed based on RAPD, SSR, AFLP, and transposon-insertion polymorphism markers^15^,^16^,^17^,^18^,^19^,^20^.

The total length of the genetic map from this study, 33,020.4 cM, was much longer than those of other reported sweetpotato maps^15^,^16^,^17^,^18^,^19^,^20^. We have considered three possibilities to explain the discrepancy although reason for the differences is not fully clear. The first is differences of genome coverage of the maps. Our map would cover the longest regions of the sweetpotato genome because of the highest density among the reported maps. The second is due to different software programs, mapping algorithms, map functions, ratios of the number of markers and populations size, and treatment of genotyping errors. All of them could explain the differences in map length as reported by the previous studies^25^,^26^. The third might be derived from residual genotyping errors. Every 1% error rate in a marker adds approximately 2 cM to the map^27^, even though substantial errors in our data were corrected with Maskov.

To date, HGs in the sweetpotato genetic maps had not been identified clearly and completely due to the shortage of duplex and triplex anchor loci in the previous marker systems, which were insufficient for saturating the maps with genetic loci, since sweetpotato has an autohexaploid genome consisting of 90 chromosomes (2n = 6x = 90). In this study, we successfully performed complete identification of the 15 HGs in sweetpotato by improving upon previous techniques as follows: (1) employing SNPs from ddRAD-Seq analysis through NGS technology to maximize genetic loci on the map, (2) using the genome sequence of *I. trifida*, a likely diploid ancestor of *I. batatas*, as a reference, which can be used to physically identify HGs, and (3) using an S1 mapping population to simplify the genetic mode for autohexaploid species.

Since SNPs are the most abundant polymorphisms across genomes, their use greatly facilitates rapid genetic analysis of autopolyploid crops with large numbers of chromosomes and complex genomes, such as sugarcane (2n = 10x = 100)^28^ and sweetpotato (this study). To identify SNPs, we mapped ddRAD-Seq reads from autohexaploid sweetpotato onto the genome sequence of *I. trifida*, a likely diploid ancestor of sweetpotato. The mapping rate of RAD-Seq reads for each progeny was approximately 70% or higher (Supplementary Table S2), indicating that the *I. trifida* genome has sufficient similarity to the sweetpotato genome to be used as a reference genome sequence. In addition, functions of the SNPs could be annotated using the *I. trifida* genome information. Approximately 90% of the SNPs were in gene regions (Supplementary Table S4), which percentage is much higher than the gene fraction of the *I. trifida* genome, 12.5%. As proposed in the previous study^23^, the combinations of restriction enzymes, *Pst*I-*Eco*RI and *Pst*I-*Msp*I, employed for the ddRAD-Seq library constructions were effective to detect gene SNPs. The ddRAD-Seq maps based on the enzymes would be biologically meaningful and beneficial for functional genomics, molecular genetics, and marker-assisted selection in breeding.

We focused only on double-simplex SNPs for linkage analysis of the S1 mapping population and eliminated other types of SNPs, e.g., duplex and triplex markers, to simplify the genetic model of the S1 mapping population for hexaploid sweetpotato (Supplementary Table S1). As a result, we successfully constructed a genetic linkage map with many double-simplex SNPs. On the other hand, it was impossible to identify HGs based on the linkage map itself, because no anchoring markers were available. Furthermore, it was also difficult to deduce the relative genetic positions of the double-simplex SNPs on different linkage maps. Indeed, in previous studies using RAPD, SSR, and AFLP markers^15^,^16^,^17^,^18^,^20^, duplex and triplex markers were used as anchors to identify HGs. However, the number of anchors from RAPD, SSR, and AFLP markers is not sufficient for identifying all HGs.

In this study, to identify a complete set of HGs, we applied another approach based on a physical mapping strategy, which differs from the previous genetic methods employing anchor markers. In principle, SNPs on the same HGs in the *I. batatas* map are basically located on a single genome sequence in diploid *I. trifida*. Based on this idea, we assigned the SNPs on the genetic maps to the genome sequence of *I. trifida*. As expected, all 15 HGs were identified, each of which had six LGs, except for four HGs consisting of seven or eight LGs (Fig. 3, Supplementary Table S5). In other words, we overcame the disadvantage of using double-simplex SNPs as anchors for polyploid genetic analysis and found that our physical mapping strategy could identify HGs in the polyploid genetic map.

In conclusion, we established the first high-density genetic map of hexaploid sweetpotato using SNP markers obtained from ddRAD-Seq analysis. This is also the first report of the identification of the complete set of 15 HGs in the sweetpotato genetic map using a physical mapping strategy. This genetic map, with a high density of SNPs, could facilitate the identification of all haplotypes of the autohexaploid genome of sweetpotato. This haplotype information would be helpful for haploid (or phased) genome sequencing in sweetpotato, meaning that it might no longer be impossible to decode the complex sweetpotato genomes completely. The molecular and genetic techniques developed in this study are also applicable to other polyploid plant species, including important crops.

## Methods

### Plant materials

A single sweetpotato cultivar, Xushu 18, the most popular cultivated line in China, was used in this study. The plants were artificially self-pollinated to obtain seeds to generate an S1 mapping population (n = 142) in NARO, Japan. Genomic DNA was extracted from leaves of individuals from this population, as well as those of the parental line, using a DNeasy Plant Mini Kit (Qiagen, Hilden, Germany).

### ddRAD-Seq analysis

Library construction and sequencing analysis of the 142 S1 lines and the parent (Xushu 18) were performed as described by Shirasawa *et al*.^23^. The ddRAD-Seq libraries were constructed with two combinations of restriction enzymes, PM and PE, and DNA fragments 300–900 bp in length were fractionated with BluePippin (Sage Science, Beverly, MA, USA). The nucleotide sequences of the libraries were determined on a HiSeq2000 system (Illumina) in single-read (101-base) or paired-end mode (93-base).

### Data processing

Primary data processing of sequence reads was performed as described by Shirasawa *et al*.^23^ with minor modifications. Low-quality sequences were removed and adapters were trimmed using PRINSEQ (version 0.20.4)^29^ and fastx_clipper in FASTX-Toolkit (version 0.0.13) (http://hannonlab.cshl.edu/fastx_toolkit). The filtered reads were mapped onto the *I. trifida* “Mx23Hm” (ITR_r1.0)^21^ genome sequence as a reference using Bowtie 2 (version 2.2.3)^30^ with parameters of maximum fragment size length, 1000 (I = 1000), in the ‘–sensitive’ preset of the ‘–end-to-end’ mode. The resulting sequence alignment/map format (SAM) files were converted to binary sequence alignment/map format (BAM) files and subjected to SNP calling using the mpileup option of SAMtools^31^ (version 0.1.19) and the mpileup2snp option of VarScan 2 (version 2.3)^32^ to obtain a variant call format (VCF) file including SNP information. The effects of SNPs on gene functions were predicted with SnpEff (version 4.2)^33^.

### SNP mining

High-confidence SNP candidates were selected using VCFtools (version 0.1.12b)^34^ with the following criteria: (i) depth of coverage ≥10 for each data point and (ii) proportion of missing data <0.25 for each locus. Using the filtered VCF files, values for read depth in genotype fields in the VCF file, i.e., DP (Quality Read Depth of bases with Phred score ≥15), RD (Depth of reference-supporting bases), and AD (Depth of variant-supporting bases), for each position were extracted across the 142 S1 individuals, and AD to DP ratios were used to calculate AAFs for each locus. SNP sites with an AAF ≥ 0.0833 and <0.2500 and those with an AAF ≥ 0.7500 and <0.9167 were selected as “AAAAAa × AAAAAa” and “Aaaaaa × Aaaaaa” double-simplex candidates, respectively.

Subsequently, the genotypes of each individual were determined as described in the Results section. In short, for “AAAAAa × AAAAAa” loci, SNPs with an AAF of 0.000 and >0.000 were determined to be homozygous REF alleles (AAAAAA) and “not homozygous” REF alleles, respectively. Likewise, for “Aaaaaa × Aaaaaa” loci, SNPs with an AAF of 1.000 were regarded as homozygous ALT alleles (aaaaaa), while the remaining SNPs with an AAF of <1.000 were considered to be “not homozygous” ALT alleles. SNPs of homozygotes versus not homozygotes fitting a 1:3 segregation ratio based on Chi-square tests (P value of ≥1%) were selected for further linkage analysis.

### Linkage analysis for map construction

The data for the selected segregating SNPs were grouped using the OneMap (version 2.0.4)^35^ via the “group” command with LOD of 7 and ordered using the “order” command (parameters of n.init = 5, subset.search = “twopt”, twopt.alg = “rcd”, THRES = 3, draw.try = TRUE, wait = 1, and touchdown = TRUE). Missing data and incorrect genotypes were then imputed with the Maskov (version 1.0)^24^ using default parameters (max error = 1; threshold = 1; column to plot = 1; data missing% = 33). The imputed data were again grouped and ordered with the OneMap using the same parameters described above. A genetic linkage map was drawn with the MapChart (version 2.2)^36^.

### Data availability

Nucleotide sequence data from the ddRAD-Seq libraries are available in the DDBJ Sequence Read Archive under accession numbers DRA004836 for PM-PE, DRA004837 for PM-SR, and DRA004838 for PE-PE. Details about the SNPs and maps are available in the Sweetpotato GARDEN database (http://sweetpotato-garden.kazusa.or.jp/).

## Additional Information

**How to cite this article:** Shirasawa, K. *et al*. A high-density SNP genetic map consisting of a complete set of homologous groups in autohexaploid sweetpotato (*Ipomoea batatas*). *Sci. Rep.* **7**, 44207; doi: 10.1038/srep44207 (2017).

**Publisher's note:** Springer Nature remains neutral with regard to jurisdictional claims in published maps and institutional affiliations.

## Supplementary Material

Supplementary Information

Supplementary Tables

## Figures and Tables

**Table 1:** Number of SNP loci and length of the genetic map based on a Xushu18 S1 population.

HGs	LGs																Total	
	1		2		3		4		5		6		7		8			
	#Locus	cM	#Locus	cM	#Locus	cM	#Locus	cM	#Locus	cM	#Locus	cM	#Locus	cM	#Locus	cM	#Locus	cM
Ib01	692	904.5	470	633.0	549	614.9	479	487.5	432	442.1	337	327.3					2,959	3,409.2
Ib02	327	599.3	473	571.1	419	475.7	320	423.4	275	319.4	253	285.6	19	33.9	26	26.3	2,112	2,734.7
Ib03	420	508.7	411	481.2	403	471.2	366	419.2	342	391.1	308	328.2					2,250	2,599.5
Ib04	383	493.1	361	483.0	363	483.0	335	413.3	387	401.8	263	249.8					2,092	2,524.0
Ib05	391	557.3	356	463.6	348	404.5	242	322.2	313	292.1	211	232.9					1,861	2,272.6
Ib06	434	520.2	287	385.7	370	351.3	318	348.5	260	334.3	222	264.6					1,891	2,204.7
Ib07	351	471.2	370	430.3	382	429.5	355	386.0	151	194.3	124	162.0	52	65.9	38	28.4	1,823	2,167.6
Ib08	464	444.5	442	438.6	312	415.4	358	341.8	213	278.6	190	217.5					1,979	2,136.4
Ib09	302	373.4	371	363.9	361	351.6	283	327.7	258	303.5	250	290.8					1,825	2,011.0
Ib10	333	391.6	330	379.9	325	375.0	341	373.5	195	244.5	226	241.3					1,750	2,005.8
Ib11	335	470.4	321	389.4	342	387.1	392	335.9	155	186.8	185	181.2	41	39.0			1,771	1,989.6
Ib12	269	462.0	318	355.1	228	342.3	302	342.2	228	245.7	199	223.1					1,544	1,970.4
Ib13	278	323.4	312	306.6	283	306.4	259	294.6	276	261.3	245	249.6					1,653	1,742.0
Ib14	411	439.7	258	380.9	316	291.6	200	230.3	170	218.8	39	56.0	48	38.8			1,442	1,656.0
Ib15	283	354.5	244	317.4	140	300.7	170	237.6	134	203.5	164	183.5					1,135	1,597.1
Total	5,673	7,313.5	5,324	6,379.9	5,141	5,999.9	4,720	5,283.6	3,789	4,317.7	3,216	3,493.4	160	177.6	64	54.7	28,087	33,020.4
