# Supplementary material for: A high-density SNP genetic map consisting of a complete set of homologous groups in autohexaploid sweetpotato (Ipomoea batatas)
Source: Sci Rep. 2017 Mar 10;7:44207. doi: 10.1038/srep44207 (PMC5345071; doi:10.1038/srep44207)
Supplement: Supplementary Information [file srep44207-s1.pdf]

Supplementary information

A high-density SNP genetic map consisting of a complete set of homologous groups in autohexaploid sweetpotato (*Ipomoea batatas*)

Kenta Shirasawa[1*], Masaru Tanaka[2], Yasuhiro Takahata[2], Daifu Ma[3], Qinghe Cao[3], Qingchang Liu[4], Hong Zhai[4], Sang-Soo Kwak[5], Jae Cheol Jeong[5], Ung-Han Yoon[6], Hyeong-Un Lee[6], Hideki Hirakawa[1], and Sachiko Isobe[1]

[1]Kazusa DNA Research Institute, Japan, [2]Kyushu Okinawa Agricultural Research Center, National Agriculture and Food Research Organization, Japan, [3]Chinese Academy of Agricultural Sciences, China, [4]China Agricultural University, China, [5]Korea Research Institute of Bioscience & Biotechnology, South Korea, and [6]Rural Development Administration, South Korea

Supplementary Table S1 Theoretical segregation ratios for the F1 and S1 progeny of autohexaploid species.

Supplementary Table S2 Number of ddRAD-Seq reads and their mapping rates for the S1 mapping progenies.

Supplementary Table S3 Genetic map based on the Xushu18 S1 population.

Supplementary Table S4 Number of sequence variations for each sequence ontology.

Supplementary Table S5 Number of SNPs mapped onto *I. trifida* scaffolds (ITR_r1.0).

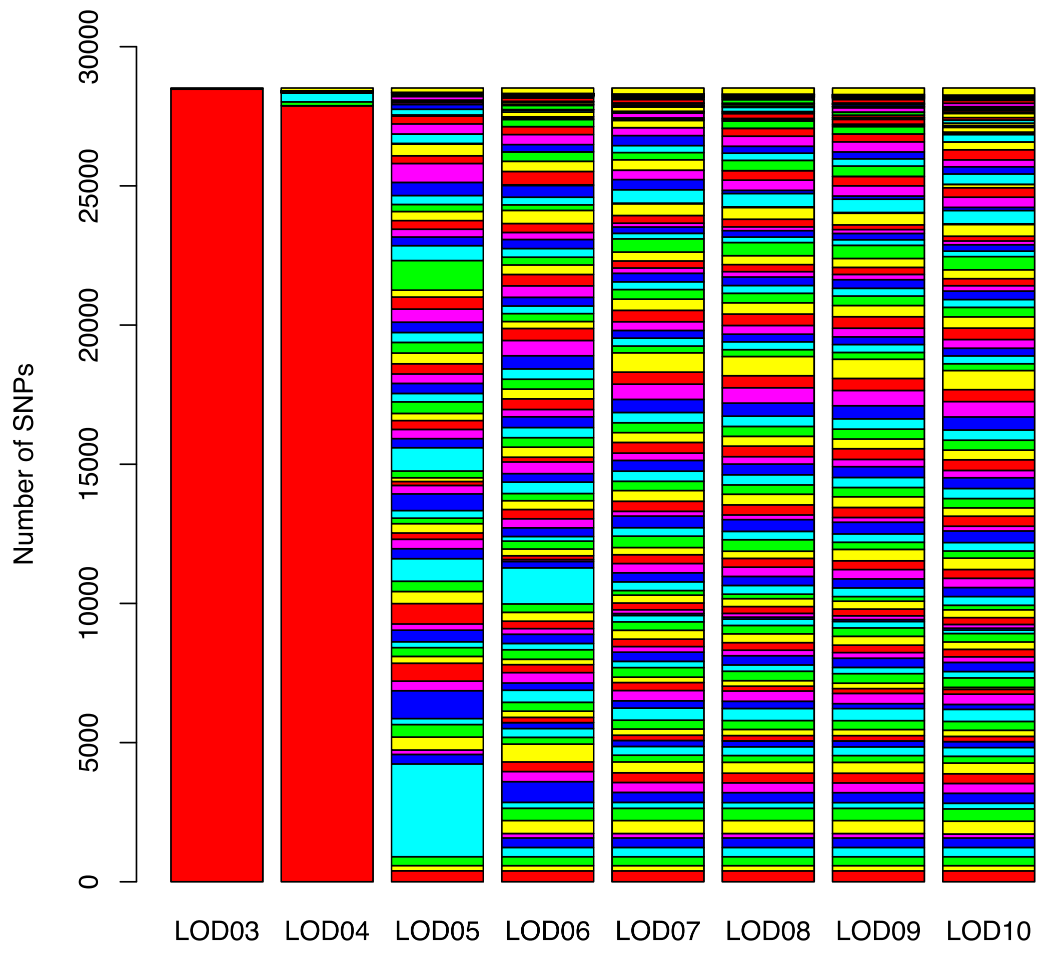

Supplementary Figure S1 Grouping of SNPs with different LOD values using the OneMap program.
